# Evaluation of gene panels for inherited cardiac disease—is less more?

**DOI:** 10.1007/s12471-019-1279-5

**Published:** 2019-04-16

**Authors:** D. Dooijes, M. Siemelink, A. F. Baas

**Affiliations:** Department of Genetics, University Medical Center Utrecht, Utrecht, The Netherlands

In this issue of the Netherlands Heart Journal, van Lint et al. describe the yield of diagnostic next-generation sequencing (NGS) gene panels for cardiomyopathies and primary arrhythmia syndromes [1]. Historically, cardiogenetic testing was performed using Sanger sequencing, usually in a gene-by-gene fashion each requiring many months. In 2012, diagnostic NGS testing was introduced in the Netherlands, enabling rapid analysis of large gene panels simultaneously (e. g. over 50 genes in 2–3 months), drastically improving cardiogenetic diagnostics.

The five cardiogenetic diagnostic centres in the Netherlands (*i.* *e.* University Medical Centre (UMC) Groningen, UMC Utrecht, Amsterdam UMC, Erasmus MC Rotterdam, UMC Maastricht) reached consensus on a 45-gene pan-cardiomyopathy core panel, which has been updated and expanded to local panels by individual centres over time, as described by van Lint et al. [1]. Their study summarises the single-centre outcome of 2,829 NGS panels analysed between 2012 and 2018. They present that in approximately half of the analysed panels a variant was detected. However, the vast majority of variants detected were so-called VUS (variants of unknown clinical significance). In only 8.8% of arrhythmia patients and 19.8% of cardiomyopathy patients pathogenic or likely pathogenic (*i.* *e.* disease-causing) variants were identified.

With more genes analysed, the amount of identified variants to be interpreted increases proportionally. Variant classification is performed by laboratory specialists (molecular geneticists) using an internationally accepted 5‑class system: Class 1. benign (no clinical significance); 2. likely benign; 3. clinical significance uncertain—VUS; 4. likely pathogenic; and 5. definitely pathogenic. Class 1 and 2 variants are not reported [2]. Variant classification involves multiple steps, including prediction algorithms, conservation analysis and checking variant frequency in control and patient databases. Along with the introduction of NGS, genetic information of control populations has rapidly emerged. In October 2014 and October 2016, the Exome aggregation consortium database (ExAC, containing over 60,000 exomes) and the Genome Aggregation Database (gnomAD, containing whole exome and whole genome data of over 140,000 individuals) respectively, became publicly available.

These databases revealed that numerous variants previously classified as pathogenic were present at population frequencies higher than the disease prevalence [3]. This indicates that previously classified variants may need reclassification. In the study of van Lint et al. variants were only incidentally reclassified. A systematic reanalysis of the 848 variants reported in 1,264 cardiomyopathy panels from our own centre during the period 2014–2018 resulted in a reclassification of 124 variants (15%) of which 117 were downgraded, mainly because of a high frequency in control populations (unpublished data).

The identification of a VUS may have several consequences. First, it requires variant interpretation by a molecular geneticist and will likely require future reclassification. Second, it may cause uncertainty for patients and relatives, which requires time-consuming genetic counselling. Third, it may lead to misinterpretation by other healthcare providers.

In an accompanying article in this issue, Christiaans et al. describe the challenges of genetic counselling in three particular cases [5]. They argue that in their experience, genetic test results, including VUS, are not difficult to understand for patients given proper counselling. This is also our experience. However, VUS are frequently misinterpreted by other healthcare providers. Therefore, pre-genetic and post-genetic counselling should be performed by well-trained physicians with considerable knowledge and experience of VUS. Christiaans et al. also highlight the importance of operating in a multidisciplinary team, consisting of cardiologists, clinical geneticists and molecular geneticists. Combining clinical and genetic information of the patient and family members in such a team facilitates the interpretation of the clinical relevance of VUS [5].

So, while proper counselling of VUS is within the realm of genetic care, a reduction of VUS would lead to more efficient and thereby improved patient care. Why are so many variants classified as VUS and is it possible to reduce this number?

Over the years there has been a tendency to increase the size of diagnostic gene panels. Identified VUS are mainly, but not exclusively, located in genes with only limited evidence of association to cardiac disease. This prompts the question whether or not these genes should be included in diagnostic gene panels.

The Clingen initiative (www.clinicalgenome.org), dedicated to building a central resource that defines the clinical relevance of genes and variants, recently published results on the curation of hypertrophic cardiomyopathy-associated genes [4]. Reviewing the literature, only 8 of the 33 hypertrophic cardiomyopathy-associated genes were reported to have definite evidence, 3 had moderate evidence and 22 had only limited evidence [4].

On the basis of our 65-gene cardiomyopathy gene panel results and literature, we identified 28 genes with limited clinical relevance. Removal of these genes would have resulted in a reduction of reported VUS of approximately 40% (519 to 315). Two of 87 likely pathogenic and 1 of 160 pathogenic variants would have been missed, but these variants (in *ABCC9, TMEM43 *and *MYOT*) were unlikely to be responsible for the (full) phenotype of the investigated patient.

Van Lint et al. report that in their fictive smaller panel of 7 hypertrophic cardiomyopathy genes, 9 of 122 pathogenic and 19 of 50 likely pathogenic variants would have been missed, a considerably larger amount. These data suggest that, while it is possible to reduce the number of VUS considerably by decreasing the number of genes in a diagnostic gene panel, it is crucial to assess the effect of each gene on overall sensitivity and specificity of the panel.

In conclusion, NGS gene panel testing has proven to be invaluable for cardiogenetic diagnostics, with more and quicker genetic diagnoses, enabling the identification of family members at risk. However, a higher mutation detection rate comes at a price of a substantial increase in the detection of VUS.

We therefore propose cardiogenetic diagnostic gene panels to be limited to genes of which there is currently sufficient evidence of association to cardiac disease. Preferably a nationwide consensus should lead to smaller diagnostic gene panels with optimal sensitivity and specificity.
